# Nuclear IL-33 amplifies NF-κB signaling to drive neutrophil recruitment and activation in the endocervical epithelium during *Mycoplasma genitalium* infection

**DOI:** 10.3389/fimmu.2026.1820874

**Published:** 2026-07-22

**Authors:** Yu Tian, Kaiming Li, Na Xie, Chi Zhang, Hao Hu, Zhemin Huang, Min Du, Ranhui Li, Jing Xie

**Affiliations:** 1 Department of Obstetrics, Affiliated Hengyang Hospital of Hunan Normal University & Hengyang Central Hospital, Hengyang, China; 2 School of Basic Medical Sciences, Hunan Normal University, Changsha, China; 3 Department of Clinical Laboratory, Affiliated Hengyang Hospital of Hunan Normal University & Hengyang Central Hospital, Hengyang, China; 4 Institute of Pathogenic Biology, Hunan Provincial Key Laboratory for Special Pathogens Prevention and Control, Hengyang Medical College, University of South China, Hengyang, China

**Keywords:** endocervical epithelial cells, IL-33, interferon regulatory factors, *Mycoplasma genitalium*, TANK-binding kinase 1

## Abstract

*Mycoplasma genitalium* has emerged as a clinically significant etiology of sexually transmitted infections that often establishes persistent infections within the female reproductive tract. Although the recruitment of neutrophils is a characteristic feature of the cervicitis associated with *M. genitalium*, the molecular mechanisms that govern this response at the mucosal interface remain incompletely defined. In this study, the infection of endocervical epithelial cells with *M. genitalium* was observed to induce a robust increase in IL-33 protein that localized within the nucleus. The transcriptional induction of the gene encoding *IL-33* was mediated by a signaling axis involving Toll-like receptor 2 (TLR2) and TANK-binding kinase 1 (TBK1), which led to the nuclear translocation and promoter binding of Interferon Regulatory Factor 1 (IRF1) and 7 (IRF7). Within the nucleus, IL-33 physically complexed with the p65 subunit of NF-κB. This interaction facilitated the recruitment and occupancy of p65 at the promoters of the genes encoding *CXCL1* and *IL-8*, thereby specifically amplifying the secretion of these chemokines. Functional assays demonstrated that the secretion of chemokines dependent on nuclear IL-33 was required for the adhesion, chemotaxis, and oxidative activation of neutrophils. These findings identify a pathogenic mechanism whereby *M. genitalium* exploits the nuclear function of IL-33 to drive chronic neutrophilic inflammation and contribute to the immunopathology of the reproductive tract.

## 1 Introduction

*Mycoplasma genitalium* constitutes a clinically significant cause of sexually transmitted infections (1, 2). In studies that screened for all three organisms within the same cohort, its prevalence rivals or exceeds those of *Chlamydia trachomatis* and *Neisseria gonorrhoeae*: in a general-population survey, *M. genitalium* prevalence fell between that of *N. gonorrhoeae* and *C. trachomatis* (3), whereas in U.S. sexually transmitted infection clinic populations it exceeded that of both (4). Unlike pathogens that cause acute symptoms, *M. genitalium* frequently establishes persistent and low grade infections that remain asymptomatic in a large proportion of infected women (5–7). This clinical presentation poses a risk because unrecognized and untreated asymptomatic infections may spread upward to the upper genital tract, leading to severe sequelae of long duration including cervicitis and pelvic inflammatory disease (8–10). Given the growing global prevalence of antimicrobial-resistant *M. genitalium* isolates (11), rising macrolide and fluoroquinolone resistance increasingly results in treatment failure and persistent infection, with a consequent risk of prolonged host inflammation and tissue damage. Therefore, delineating the host signaling pathways that drive mucosal inflammation is critical, as it may identify novel host-directed therapeutic targets to complement traditional antimicrobial therapy.

The primary interface for *M. genitalium* infection is the cervical epithelium (12). Although *M. genitalium* lacks a cell wall, the bacterium possesses a complex array of lipoproteins that act as potent agonists for Toll-like receptors (TLRs), particularly TLR2 and TLR6 (13–16). Cervical epithelial cells serve as frontline innate sentinels and engage pattern-recognition receptors to sense microbial challenge (17). To study these interactions, researchers utilize various *in vitro* models such as the End1/E6E7 endocervical epithelial cell line which retains the phenotypic characteristics of the primary tissue (18). Upon stimulation, these cells secrete specific proinflammatory mediators that initiate the recruitment of immune cells (12). IL-33 belongs to the IL-1 cytokine family and is an important mediator of mucosal immunity, shaping local inflammatory responses at epithelial barriers (19).

IL-33 is classically characterized as a soluble alarmin released from cells undergoing necrosis, thereby initiating immune activation (20). This function has been well-documented across diverse pathological contexts. In allergic asthma, extracellular IL-33 promotes ILC2 activation and drives eosinophil-dominant inflammation (21); in parasitic infections, it promotes the expulsion of helminths by boosting Th2 immunity (22); and in mechanical tissue injury, it signals for tissue repair and remodeling (23). However, beyond its extracellular role, IL-33 possesses a distinct nuclear biology. IL-33 harbors a nuclear localization motif together with a chromatin-associating region, enabling it to reside in the nucleus where it can modulate transcriptional activity (24, 25). While this nuclear function remains less understood than the extracellular role, evidence suggests the regulation of inflammation occurs intracellularly through interaction with transcription factors such as NF-κB (25).

In the context of *M. genitalium* infection, which typically involves adherence or invasion without immediate lysis, the role of IL-33 remains undefined. In this study, we identify a pathogenic mechanism whereby *M. genitalium* infection induces full-length IL-33 that remains localized in the nucleus and does not function as a secreted cytokine. We demonstrate that this nuclear IL-33 (nIL-33) associates with the NF-κB p65 subunit and serves as a transcriptional co-regulator, thereby enhancing the secretion of the neutrophil-recruiting chemokines CXCL1 and IL-8. These findings elucidate a mechanism by which a pathogen manipulates nuclear signaling to drive the neutrophilic inflammation characteristic of cervicitis associated with *M. genitalium* and offer mechanistic insight into the inflammatory immunopathology linked to infertility.

## 2 Materials and methods

### 2.1 Reagents and antibodies

*M. genitalium* strain G37 (33530) was obtained from the ATCC. The immortalized human endocervical epithelial cell line End1/E6E7 (ATCC CRL-2615) was cultured as previously described (18). Specific pharmacological inhibitors included: the TLR2 inhibitor C29 (MedChemExpress); the TLR4 signaling inhibitor TAK-242 (CLI-095, InvivoGen); and the specific TBK1/IKKϵ inhibitor BX795 (InvivoGen). Primary antibodies against IL-33 (sc-517600), IRF1 (sc-514544), IRF7 (sc-74472), and p65 (sc-8008), used for WB, IP, and ChIP, were obtained from Santa Cruz Biotechnology. For signaling and loading controls, we utilized the following antibodies from Cell Signaling Technology (CST): anti-phospho-TBK1 (Ser172) (D52C2, WB), anti-total TBK1 (D1B4, WB/IP), and antibodies for Histone H3 (D1H2), GAPDH (D16H11), and β-actin (13E5). Additionally, the neutralizing antibody against human TLR2 (clone TL2.1; WB/IP) was purchased from BioLegend.

### 2.2 *Mycoplasma* culture, heat-inactivation, and lipoprotein extraction

*M. genitalium* strain G37 was grown in SP-4 broth as previously described (26), with active growth monitored by the colour change of SP-4 broth from red to orange-yellow. Cultures were harvested at late-log phase, and the titre of each batch was established by serial dilution and plating on SP-4 agar, with colony-forming units (CFU) enumerated after 7–14 days of incubation; the remainder of each titred culture was then cryopreserved with cryoprotectant as single-use aliquots at −80 °C. Only batches with a consistent titre (typically 1–2 × 10^7^ CFU/mL) were used. On the day of infection, an aliquot was thawed and the multiplicity of infection (MOI) was set as CFU per End1/E6E7 cell from the pre-established titre of that batch (27). Because the slow growth of this fastidious organism precludes same-day colony enumeration, MOI was defined from this pre-titred frozen stock, and the applied dose was confirmed retrospectively by *mgpB* qPCR as described previously (28). For each experiment, an aliquot of the thawed bacterial suspension was stained with LIVE/DEAD™ BacLight™ kit (Molecular Probes) to assess viability; only preparations with >90% live bacteria were used. For heat-killed *M. genitalium* (HK-Mg), bacterial pellets were rinsed three times in sterile PBS and then heated at 100°C for 15 min to achieve complete inactivation. Membrane lipoproteins (LP) were isolated by Triton X-114-based temperature-dependent phase separation following published protocols (29). Briefly, mycoplasma pellets were lysed in 1% Triton X-114 at 4°C for 2 h, followed by phase separation at 37°C. The detergent-rich phase containing the lipoproteins was washed, ethanol-precipitated, and resuspended in PBS. Protein concentrations were quantified by BCA assay, and all extracts were confirmed to be free of viable bacteria by re-culturing in SP-4 broth.

### 2.3 Bacterial infection and stimulation assays

The immortalized human endocervical epithelial cell line End1/E6E7 was cultured in keratinocyte serum-free medium (K-SFM) supplemented with 0.1 ng/mL recombinant human EGF and 50 μg/mL bovine pituitary extract. For live infection, End1/E6E7 cells were seeded in antibiotic-free K-SFM 24 h prior to challenge. Infection experiments were performed at the indicated multiplicities of infection (MOI 10, 50, or 100). Post-infection supernatants and cell lysates were harvested at specific time points for downstream cytokine quantification and molecular analysis.

### 2.4 Quantification of cell-associated *M. genitalium*

Cell-associated *M. genitalium* was quantified by quantitative PCR targeting the *mgpB* (MgPa) gene. At the indicated time points, culture supernatants were removed and End1/E6E7 monolayers were washed three times with PBS to remove non-adherent organisms. Cells were then detached by trypsinization and total DNA was extracted with DNeasy Blood & Tissue Kit (Qiagen, Hilden, Germany). *mgpB* copy number was determined by qPCR on LightCycler 96 instrument (Roche Diagnostics, Mannheim, Germany) using primers as described previously (28), and interpolated from a standard curve generated from a quantified synthetic double-stranded DNA (gBlock) standard. For burden-control experiments, cells were treated with inhibitors or transfected with siRNA/shRNA exactly as in the corresponding signaling experiments prior to infection, and cell-associated *mgpB* was compared across conditions. For the persistence assay, cell-associated *mgpB* was measured at 0, 12, 24 and 48 h post-infection.

### 2.5 RNA interference

To achieve transient silencing of *TBK1* and *IL-33*, cells were introduced with target-specific siRNAs or a non-targeting scrambled negative control (Santa Cruz Biotechnology). siRNA delivery was carried out with Lipofectamine RNAiMAX (Invitrogen) following the supplier’s recommended protocol. For the knockdown of transcription factors IRF1 and IRF7, short hairpin RNA (shRNA) plasmid vectors (TRC library, Sigma-Aldrich) were transfected using Lipofectamine 3000 (Invitrogen). Knockdown efficiency was verified by immunoblotting 48 hours post-transfection before infection challenges.

### 2.6 Quantitative real-time PCR

Total RNA was isolated from cultured cells with TRIzol™ Reagent (Invitrogen) following standard procedures (30). First-strand cDNA was generated using the PrimeScript™ RT Reagent Kit with gDNA Eraser (Takara). qPCR was performed on an Applied Biosystems 7500 Real-Time PCR platform with TB Green^®^ Premix Ex Taq™ II (Takara). Transcript abundance of *IL-33*, *CXCL1*, and *IL-8* was determined by the 2^-ΔΔCt^ method and normalized against GAPDH as the internal reference (31).

### 2.7 Enzyme-linked immunosorbent assay

CXCL1, IL-8, and IL-33 concentrations in clarified culture supernatants were determined with DuoSet^®^ ELISA Development Systems (R&D Systems) in accordance with the supplier’ s instructions. Absorbance values were recorded at 450 nm and corrected by reference readings at 570 nm using a Synergy H1 plate reader (BioTek).

### 2.8 Subcellular fractionation and immunoblotting

To analyze nuclear and cytoplasmic protein distribution, cells were subjected to nuclear–cytoplasmic fractionation with NE-PER™ Nuclear and Cytoplasmic Extraction Reagents (Thermo Fisher Scientific) supplemented with a protease/phosphatase inhibitor cocktail (Roche) following the supplier’s recommended protocol. Comparable protein amounts were resolved by SDS–PAGE and electrotransferred to PVDF membranes. Immunoblotting was performed using specific primary antibodies and secondary antibodies conjugated to horseradish peroxidase. The protein bands were visualized using enhanced chemiluminescence reagents (32).

### 2.9 Co-Immunoprecipitation

Nuclear extracts were pre-cleared with Protein A/G Magnetic Beads (Thermo Fisher Scientific) and then incubated with 2 µg of anti-IL-33, anti-p65, anti-TLR2, anti-TBK1, or isotype control IgG antibodies as described previously (33). Fresh magnetic beads were added for 2 h to capture the immune complexes. Beads were washed five times with low-salt lysis buffer, and bound proteins were eluted by boiling in 2× SDS loading buffer for immunoblot analysis.

### 2.10 Chromatin immunoprecipitation

ChIP assays were conducted using the SimpleChIP^®^ Enzymatic Chromatin IP Kit (Cell Signaling Technology) following the manufacturer’s protocol. Briefly, cells were cross-linked with 1% formaldehyde for 10 min, and chromatin was digested with micrococcal nuclease to ~150–900 bp fragments. Immunoprecipitation was carried out with ChIP-validated antibodies against IRF1, IRF7, p65, IL-33, or normal rabbit IgG (negative control). After reversal of cross-linking, purified DNA was analyzed by qPCR using primers specific for the *IL-33*, *CXCL1*, and *IL-8* promoters. Primer sequences, amplicon coordinates (relative to TSS), product lengths, and negative control regions are detailed in **Supplementary Table 1**. Enrichment was calculated as a percentage of input.

### 2.11 Dual-luciferase reporter assay

To assess cooperative transcriptional transactivation between IL-33 and NF-κB, End1/E6E7 cells were plated in 24-well format and transiently cotransfected with an NF-κB-dependent firefly luciferase reporter plasmid (NF-κB-Luc, 200 ng), an internal control pRL-TK (20 ng), and plasmids encoding human p65 and/or full-length IL-33 proteins (100 ng each) via Lipofectamine 3000. All reporter experiments were performed without *M. genitalium* exposure to determine the intrinsic synergy of the ectopically expressed proteins. After 24 h, activities were measured using the Dual-Luciferase^®^ Reporter Assay System (Promega). Results are expressed as relative luciferase units (RLU) normalized to the Renilla internal control.

### 2.12 HL-60 cell culture and differentiation

The HL-60 promyelocytic leukemia cell line (ATCC) was maintained in RPMI 1640 supplemented with 10% FBS. To generate chemotaxis-competent neutrophil-like derivatives (dHL-60), HL-60 cells were exposed to 1.3% DMSO (Sigma-Aldrich) for 5 days. Differentiation was confirmed by assessment of nuclear morphology and surface CD11b expression as described previously (34, 35).

### 2.13 Neutrophil functional assays

Chemotaxis of differentiated HL-60 cells (used as a model for primary neutrophils) was quantified using the CellTiter-Glo Luminescent Cell Viability Assay (Promega) in a Transwell system to objectively measure migration without manual counting bias (36). Luminescence (RLU) was recorded on a Synergy H1 microplate reader (BioTek). Adhesion of dHL-60 to human endothelial monolayers was quantified using a calcein-AM fluorescence assay as described previously (37). Activation of dHL−60 cells was assessed by measuring cytochrome c reduction (superoxide dismutase-inhibitable) and the cleavage of the chromogenic substrate MeO-Suc-AAPV-pNA for neutrophil elastase, following established protocols (38, 39).

### 2.14 Statistical analysis

Data are presented as mean ± SEM of at least three independent biological replicates (each replicate represents a separately cultured batch of End1/E6E7 cells). Comparisons among multiple groups were performed using one-way ANOVA followed by Tukey’s *post-hoc* test for pairwise comparisons. For comparisons involving a single control group (e.g., all groups vs. uninfected), Dunnett’s *post-hoc* test was used. A p-value < 0.05 was considered statistically significant.

## 3 Results

### 3.1 *Mycoplasma genitalium* infection induces nuclear accumulation of IL-33 and secretion of CXCL1 and IL-8

To characterize the innate immune response of the endocervix to *M. genitalium*, End1/E6E7 endocervical epithelial cells were infected with *M. genitalium* strain G37 at varying MOI. We first confirmed that the organism establishes a stable, cell-associated population in these cells: cell-associated bacterial genome copies (mgpB qPCR on PBS-washed monolayers) were negligible immediately after inoculation (0 h), rose to a plateau by 12 h, and were maintained without a significant change through 48 h (**Supplementary Figure 1**). The absence of a significant increase between 12 and 48 h indicates that *M. genitalium* persists as a stable cell-associated population over the experimental window rather than being cleared, consistent with its reported capacity to establish persistent infection of endocervical epithelial cells (40).

**Figure 1:**
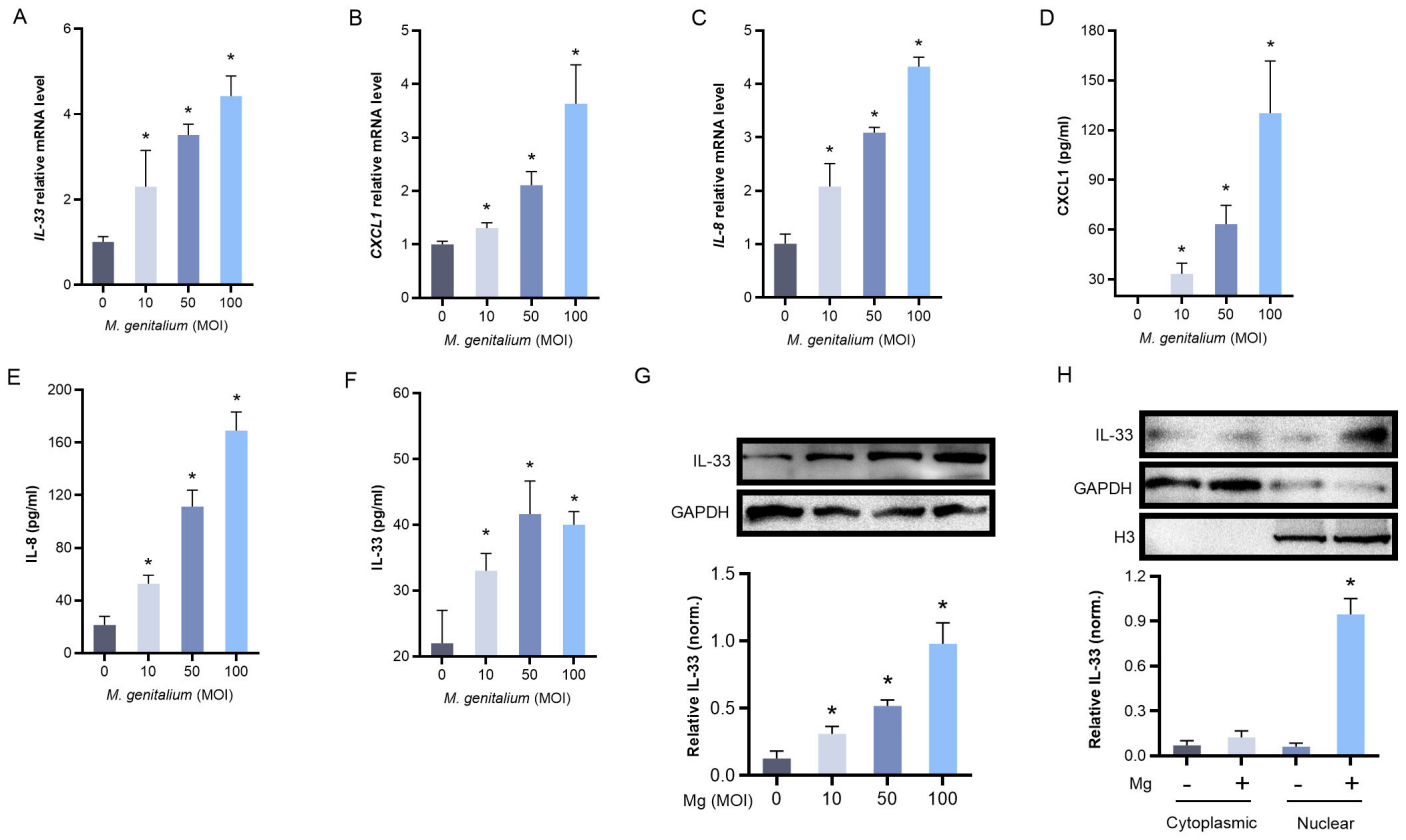
*Mycoplasma genitalium* infection induces nuclear retention of IL-33 and secretion of CXCL chemokines. **(A–C)** End1/E6E7 cells were infected with *M. genitalium* (MOI 10, 50, 100) for 24 h. mRNA levels of *IL-33* **(A)**, *CXCL1* **(B)**, and *IL-8* **(C)** were analyzed by qPCR. **(D–F)** Protein levels of CXCL1 **(D)**, IL-8 **(E)**, and IL-33 **(F)** in the culture supernatants were measured by ELISA at the indicated MOI. **(G)** Immunoblot analysis of IL-33 in whole-cell lysates (WCL) from cells infected at MOI 0, 10, 50 and 100 for 24 h. **(H)** Subcellular fractionation of cells infected with *M. genitalium* (MOI 100) for 24 h, followed by immunoblotting for IL-33. Histone H3 and GAPDH served as nuclear and cytoplasmic loading controls, respectively. Band intensities were quantified by densitometry. Nuclear IL−33 was normalized to Histone H3; cytoplasmic IL−33 was normalized to GAPDH. Data are mean ± SEM of three independent biological experiments (n=3), *p < 0.05 vs uninfected control.

We next characterized the epithelial response to infection. Infection induced a robust, dose-dependent upregulation of *IL-33*, *CXCL1*, and *IL-8* mRNA (**Figures 1A–C**). Consistent with the transcriptional data, ELISA analysis of culture supernatants confirmed significantly elevated secretion of CXCL1 and IL-8 (**Figures 1D, E**). However, despite high levels of mRNA, only minimal amounts of IL-33 protein were detected in the supernatants at all time points tested (**Figure 1F**). Immunoblotting of whole-cell lysates revealed a significant increase in full-length IL-33 protein abundance (**Figure 1G**). To resolve the discrepancy between intracellular induction and lack of secretion, we performed subcellular fractionation. While CXCL1 and IL-8 were secreted, the induced IL-33 protein was found predominantly in the nuclear fraction, co-segregating with the nuclear marker Histone H3 rather than the cytoplasmic marker GAPDH (**Figure 1H**). Validation of subcellular fractionation purity showed that Histone H3 was confined to the nuclear fraction and GAPDH to the cytoplasmic fraction, with <5% cross−contamination (**Supplementary Figure 2**). Importantly, LDH release assays showed no significant cytotoxicity at MOI 100 (**Supplementary Figure 3**), confirming that cell membrane integrity was maintained during infection. Thus, the minimal IL-33 detected in the supernatant is unlikely to result from passive leakage of damaged cells. These data indicate that *M. genitalium* infection induces IL-33 that is predominantly retained in the nucleus, with only minor amounts detected in the cytoplasm or supernatant, distinguishing this response from its classical role as a secreted alarmin.

### 3.2 TLR2–TBK1 axis mediates the transcriptional induction of *IL-33* mRNA

**Figure 2:**
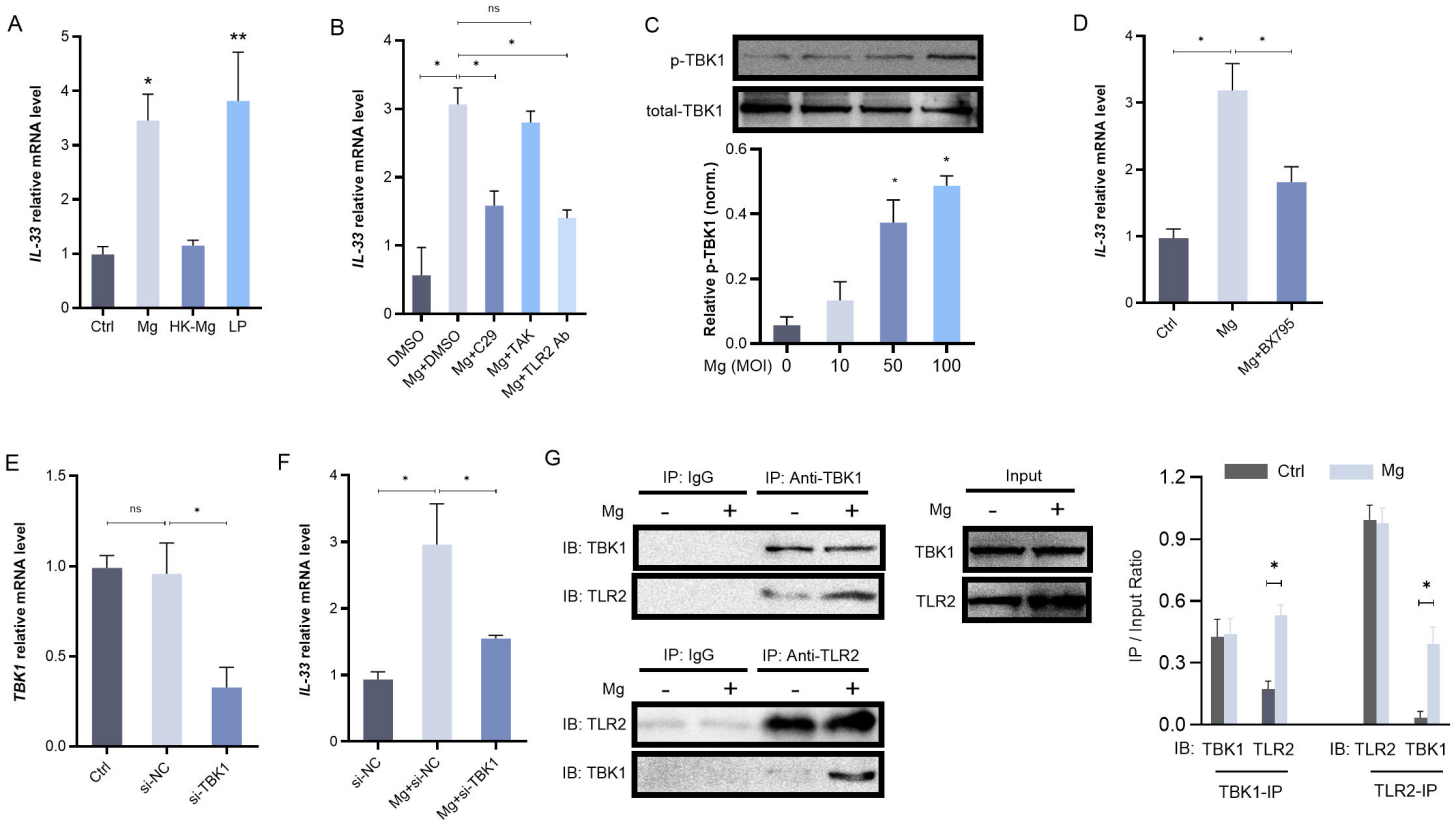
TLR2/TBK1 signaling drives *IL-33* mRNA expression. **(A)** *IL-33* mRNA expression in cells stimulated with *M. genitalium* (100 MOI), heat-killed *M. genitalium* (HK-Mg, equivalent to MOI 100), or purified membrane lipoproteins (LP, 10μg/ml). **(B)** Cells were pretreated with anti-TLR2 Ab (10μg/ml), C29 (50μmol/L), or TAK-242 (1μmol/L) for 1h prior to infection; *IL-33* levels were measured by qPCR. DMSO (0.1%) employed as the vehicle control. **(C)** Immunoblot of p-TBK1 (Ser172) and total TBK1 kinetics following infection. **(D)** Cells were treated with the TBK1 inhibitor BX795 (10 μmol/L) and *IL-33* mRNA was quantified by qPCR. **(E)** End1/E6E7 cells were transfected with TBK1 siRNA, and the knockdown efficiency was assessed by qPCR. **(F)** *IL-33* mRNA levels were measured in TBK1−depleted cells by qPCR. **(G)** Co-IP of TLR2 and TBK1 from cell lysates infected with *M. genitalium*. Data are mean ± SEM of three independent biological experiments (n=3). *p < 0.05, **p < 0.01 as compared with the uninfected control **(A)** or indicated groups **(B, D–G)**. ns, no significant difference between groups **(B, E)**.

We next investigated the upstream signaling pathways governing *IL-33* induction. Stimulation of End1/E6E7 cells with membrane lipoprotein derived from *M. genitalium*, but not heat-killed bacteria, recapitulated the transcriptional induction of *IL-33* mRNA observed with live infection, suggesting that membrane lipoproteins are likely a key pathogen-associated molecular pattern responsible for this response (**Figure 2A**). Blocking studies using a TLR2 neutralizing antibody or the specific inhibitor C29 significantly attenuated *IL-33* mRNA expression, whereas TLR4 inhibition (TAK-242) had no effect (**Figure 2B**). Given the role of TBK1 in integrating innate immune signals, we examined its activation status. As shown in **Figure 2C**, infection with *M. genitalium* at MOI 50 or 100 for 30 minutes induced robust phosphorylation of TBK1 at Ser172, indicating rapid activation of TBK1 in response to the pathogen. Pharmacological inhibition of TBK1 using BX795, or siRNA-mediated knockdown of TBK1, markedly reduced infection-induced *IL-33* upregulation (**Figures 2D–F**). Furthermore, co-immunoprecipitation assays showed that TLR2 and TBK1 co-immunoprecipitated, and this association was enhanced upon *M. genitalium* infection (**Figure 2G**). Consistent with a lipoprotein-driven mechanism, purified membrane lipoprotein induced TBK1 phosphorylation at Ser172 in a TLR2-dependent manner, as this induction was attenuated by C29 (**Supplementary Figure 4**). None of the inhibitors (C29, BX795, TAK-242) or TBK1 silencing significantly altered the cell-associated *M. genitalium* burden relative to infected controls (**Supplementary Figures 5A, B**), indicating that these changes in IL-33 induction reflect altered host signaling rather than differences in bacterial load. Collectively, these findings suggest a model in which *M. genitalium* activates TLR2−dependent signaling, partly through its membrane lipoproteins, leading to TBK1 activation and subsequent *IL-33* transcription; in this model, TLR2 and TBK1 are recruited into the same infection-enhanced signaling complex, although the exact adaptor proteins linking them remain to be identified.

### 3.3 IRF1 and IRF7 Are Required for TBK1-dependent transcriptional induction of *IL-33*

**Figure 3:**
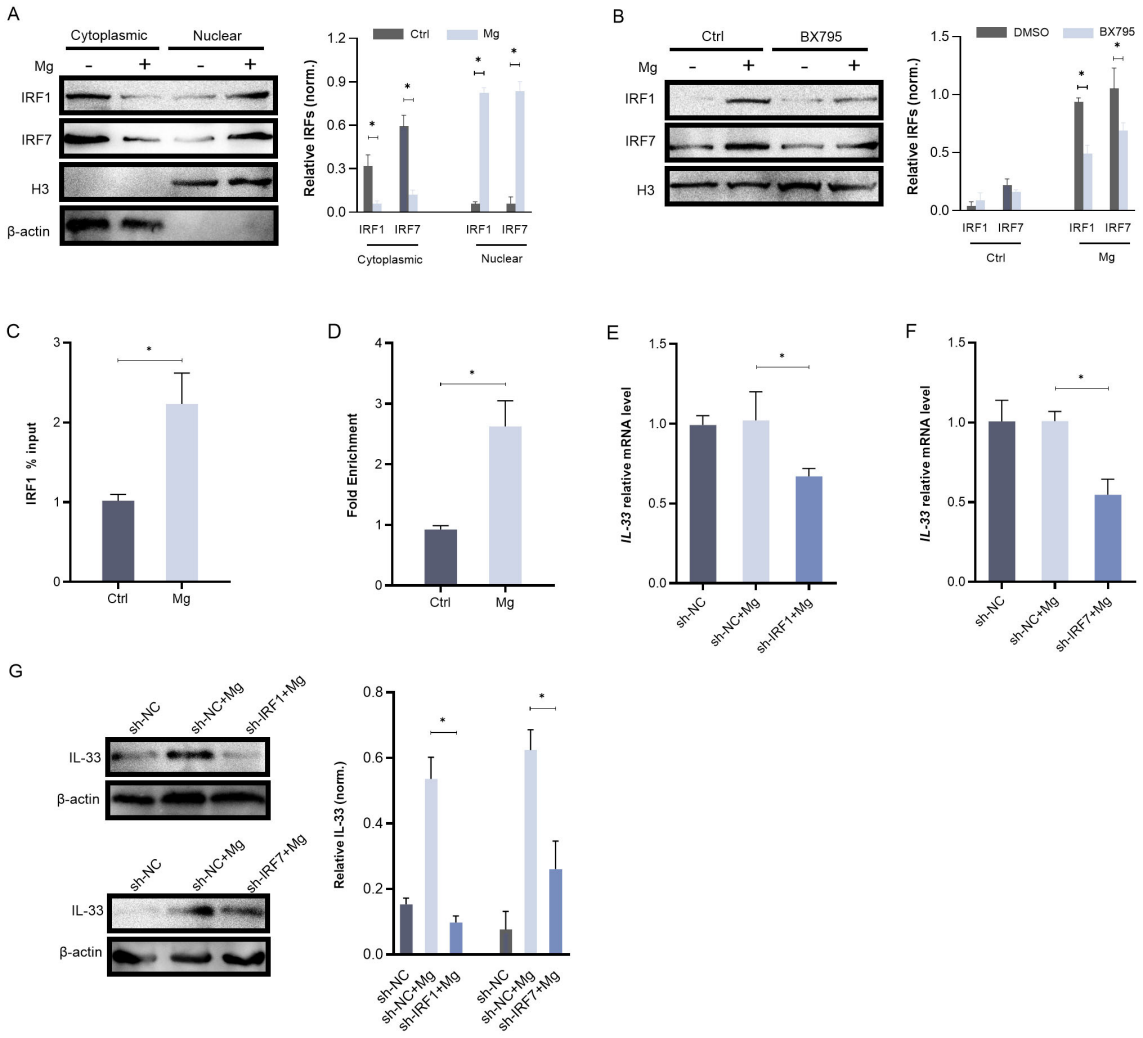
IRF1 and IRF7 are transcriptional regulators of *IL-33*. **(A)** Immunoblot of IRF1 and IRF7 in nuclear fractions of cells infected with *M. genitalium* for 24 h. **(B)** Impact of TBK1 inhibitor BX795 on the nuclear accumulation of IRF1 and IRF7, DMSO (0.1%) employed as the vehicle control. **(C, D)** ChIP-qPCR analysis of IRF1 and IRF7 binding to the *IL-33* promoter. Data are expressed as the percentage of input DNA (% Input). **(E–G)** End1/E6E7 cells were transfected with shRNA targeting IRF1 or IRF7, followed by *M. genitalium* infection. *IL-33* mRNA and protein levels were assessed by qPCR **(E, F)** and immunoblot **(G)**. Data are mean ± SEM of three independent biological experiments (n=3). *p < 0.05 vs. indicated groups **(C–F)**.

Bioinformatic analysis of the *IL-33* promoter revealed potential Interferon-Stimulated Response Elements (ISREs). We examined the role of Interferon Regulatory Factors (IRFs). Subcellular fractionation showed that *M. genitalium* infection induced the nuclear translocation of both IRF1 and IRF7 (**Figure 3A**). This translocation was blocked by TBK1 inhibition (**Figure 3B**) or siRNA knockdown (**Supplementary Figure 6**). ChIP-qPCR confirmed that infection significantly enriched the binding of IRF1 and IRF7 to the specific consensus sequences within the *IL-33* promoter (**Figures 3C, D**). Functional relevance was confirmed using RNA interference (**Supplementary Figure 7**); Silencing of either IRF1 or IRF7 partially reduced the *M. genitalium*-induced upregulation of *IL-33* mRNA and protein (**Figures 3E–G**). This silencing did not significantly change the cell-associated *M. genitalium* burden (**Supplementary Figure 5C**), confirming that the reduced IL-33 induction was not secondary to an altered bacterial load. These results indicate that IRF1 and IRF7 contribute to and amplify the TLR2/TBK1-dependent induction of IL-33.

### 3.4 Nuclear IL-33 associates with NF−κB in a nuclear complex and enhances p65−driven transcriptional activation

**Figure 4:**
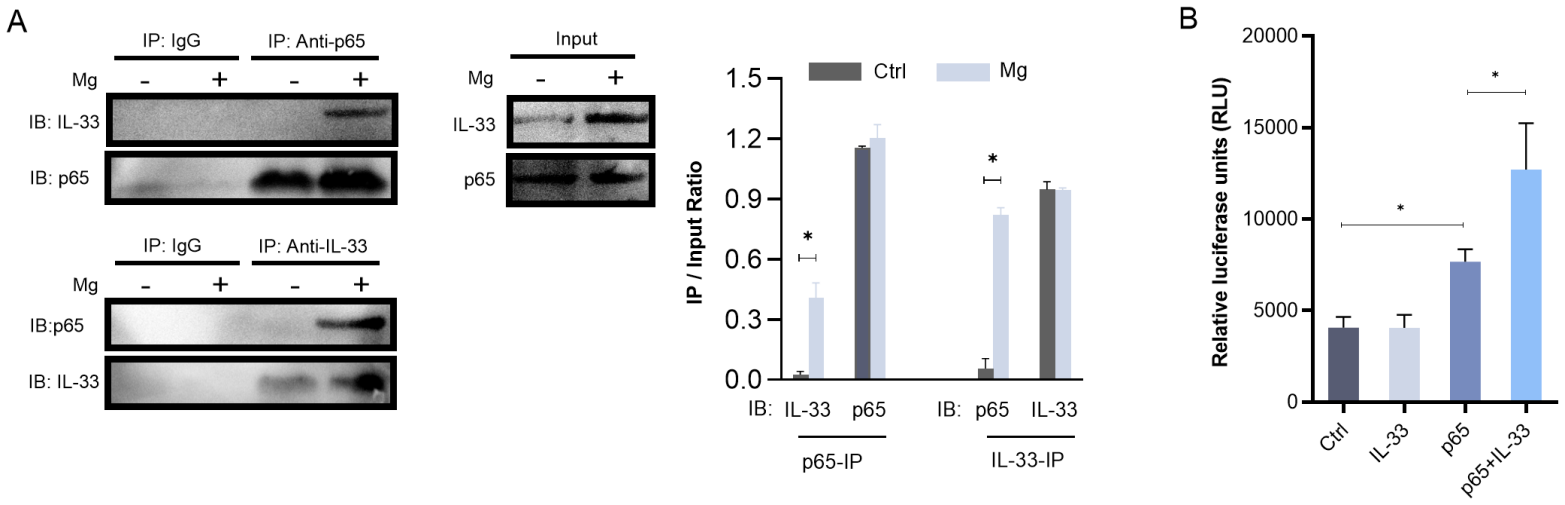
Nuclear IL-33 physically complexes with p65 to potentiate its transcriptional activity. **(A)** Nuclear extracts from *M. genitalium*-infected cells were subjected to Co-IP using anti-p65 (upper panel) or anti-IL-33 antibody (lower panel) antibodies, followed by immunoblotting for the indicated partners. IgG served as a negative control. **(B)** End1/E6E7 cells were co-transfected with NF-κB-Luc, pRL-TK, and plasmids encoding human *IL-33* and p65 proteins as described in Materials and Methods. Data represent normalized RLU and are shown as mean ± SEM of three independent biological experiments (n=3). *p < 0.05 vs. indicated group.

Given the nuclear retention of IL-33, we hypothesized it functions as a transcriptional regulator rather than a cytokine. We first investigated its interaction with the NF-κB subunit p65. Co-IP using nuclear extracts from infected cells showed that endogenous IL-33 and p65 co-exist in the same nuclear complex (**Figure 4A**). To confirm that the infection-induced nIL-33 is full-length rather than a truncated or alternatively spliced isoform, nuclear fractions from uninfected and infected cells were immunoblotted with antibodies recognizing distinct N-terminal and C-terminal IL-33 epitopes. Both antibodies detected a single ~31 kDa band in the infected nuclear fraction, corresponding to full-length IL-33 (**Supplementary Figure 8**). Having established the identity of the endogenous nIL-33, we next performed NF-κB-responsive luciferase reporter assays to test whether this complex enhances p65-driven transcription. For these assays, we utilized a plasmid expressing full-length IL-33 (which retains its endogenous NLS), thereby mirroring the nuclear accumulation observed during infection. Co−expression of IL−33 with p65 significantly potentiated p65−mediated transactivation (**Figure 4B**), whereas IL−33 alone had no effect. Together, these biochemical and functional assays suggest that nIL-33 serves as a transcriptional co-activator, forming a functional complex with p65 to enhance NF-κB signaling.

### 3.5 Nuclear IL-33 is essential for p65 occupancy at *CXCL1* and *IL-8* promoters

**Figure 5:**
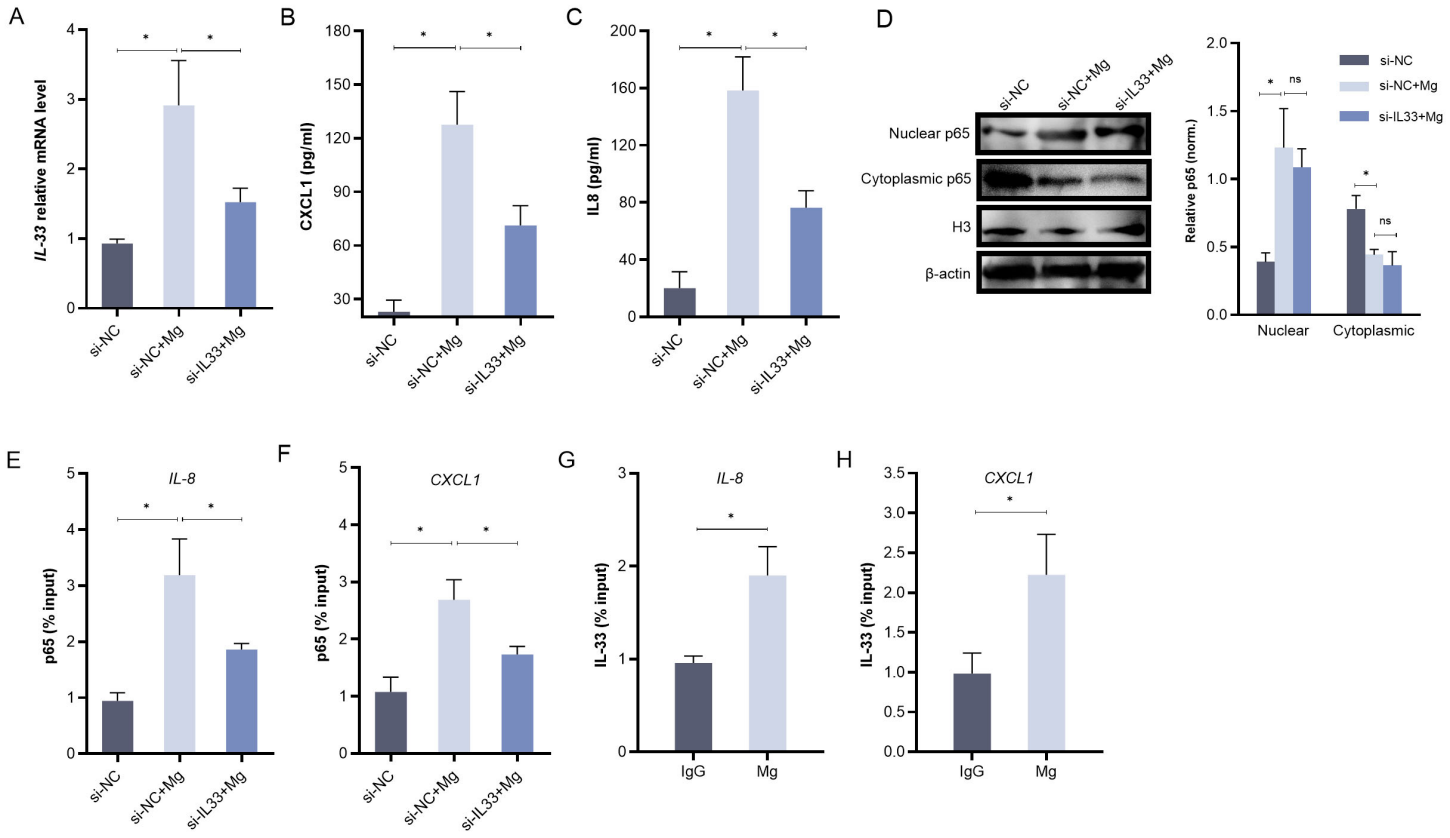
Nuclear IL-33 is required for p65 recruitment to chemokine promoters. **(A)** qPCR validation of *IL-33* knockdown efficiency at 48 h post-transfection. **(B, C)** CXCL1 **(B)** and IL-8 **(C)** secretion in cells transfected with control (si-NC) or *IL-33* siRNA (si-IL33) followed by *M. genitalium* infection. Secreted protein levels in culture supernatants were quantified by ELISA. **(D)** Subcellular distribution of p65. Representative immunoblots of nuclear (N) and cytoplasmic (C) fractions. Histone H3 and β-actin served as nuclear and cytoplasmic loading controls, respectively. Note the intact nuclear translocation of p65 despite *IL-33* silencing. **(E, F)** ChIP-qPCR analysis demonstrating that p65 occupancy at the *CXCL1* and *IL−8* promoters is significantly compromised upon *IL-33* depletion. Data are expressed as % Input. **(G,H)** ChIP-qPCR confirming the physical recruitment of IL-33 to the same regulatory regions of *CXCL1* and *IL−8*. Data are mean ± SEM of three independent biological experiments (n=3). *p < 0.05 vs. indicated group. ns, no significant difference between groups **(D)**.

To determine if the *de novo* synthesized nIL-33 was required for this response, we silenced endogenous *IL-33* using high-potency siRNA prior to *M. genitalium* infection (**Figure 5A**; **Supplementary Figure 9**). *IL-33* knockdown resulted in a profound reduction in CXCL1 and IL-8 secretion (**Figures 5B, C**). Subcellular fractionation and immunoblotting demonstrated that p65 nuclear translocation was not substantially affected in *IL-33*-depleted cells following infection (**Figure 5D**), indicating that nIL-33 operates downstream of nuclear entry. However, ChIP-qPCR assays revealed that in *IL-33*-depleted cells, p65 occupancy at the specific κB sites of the *CXCL1* and *IL-8* promoters was significantly diminished (**Figures 5E, F**). Reciprocal ChIP assays further demonstrated inducible recruitment of IL−33 itself to these same regulatory loci during infection (**Figures 5G, H**). IL-33 silencing did not alter the cell-associated *M. genitalium* burden (**Supplementary Figure 5B**), excluding a change in bacterial load as the basis for the reduced chemokine response. Together, these data indicate that nIL-33 promotes p65 binding to the *CXCL1* and *IL-8* promoters, thereby enhancing chemokine gene expression.

### 3.6 The IL-33/CXCL axis mediates neutrophil−like cell adhesion, chemotaxis, and activation

**Figure 6:**
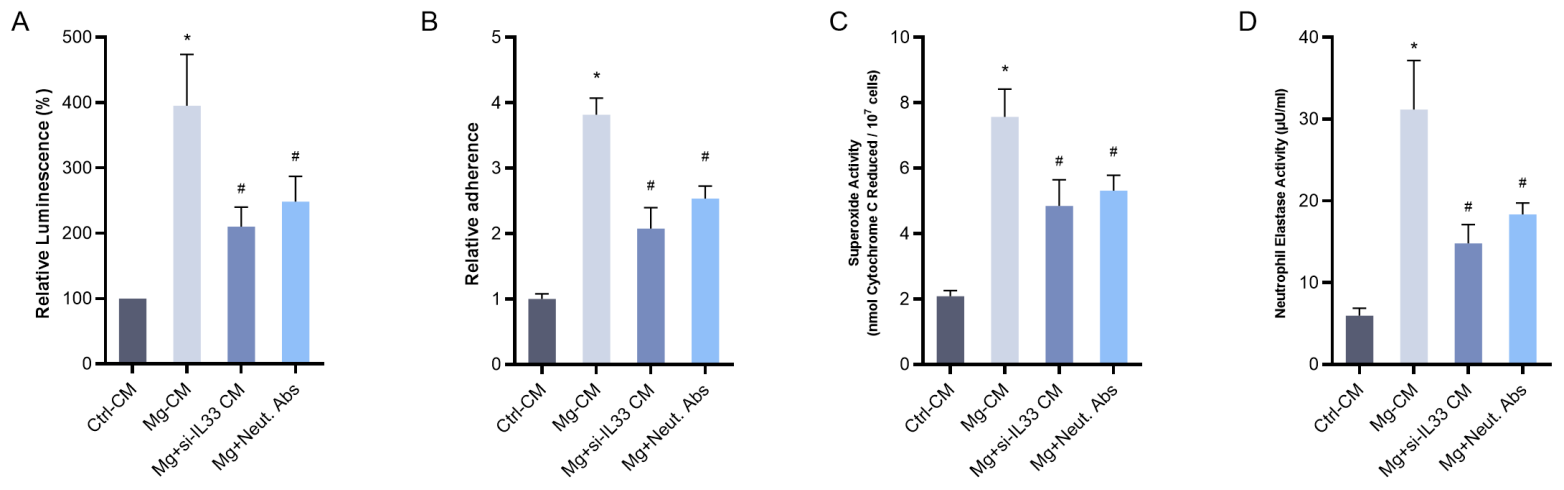
The IL-33/CXCL axis orchestrates neutrophil-like cell recruitment and activation. Conditioned media from *M. genitalium*-infected (Mg-CM), or *M. genitalium*-infected/*IL-33*-silenced End1/E6E7 cells were applied to dHL-60 cells, a neutrophil-like cell line. “Neut. Abs” indicates CM treated with a neutralizing antibody cocktail against CXCL1 and IL-8 (2 μg/mL each). **(A)** Chemotaxis was quantified using a luminescence−based ATP viability assay. Data are expressed as percentage of luminescence relative to control CM (set to 100%). **(B)** Adhesion of calcein-AM–labeled dHL-60 to endothelial monolayers, Data are expressed as relative adherence normalized to the control CM (1.0). **(C)** Superoxide activity in dHL-60 cells measured by SOD-inhibitable cytochrome c reduction, expressed as nmol Cytochrome c reduced per 10^7^ cells. **(D)** Neutrophil Elastase Activity in supernatants quantified by the cleavage of MeO-Suc-AAPV-pNA, expressed in μU/ml. Data are mean ± SEM of three independent experiments (n=3). *p < 0.05 vs. Ctrl-CM, ^#^p < 0.05 vs. Mg-CM.

To determine the biological significance of the epithelial signaling axis, we assessed the capacity of the endocervical secretome to orchestrate neutrophil−like functions using differentiated HL−60 (dHL−60) cells as a model for primary neutrophils. As shown in **Figure 6A**, conditioned medium (CM) from infected cells elicited a robust increase in dHL−60 chemotaxis compared with control-CM as measured by luminescence. This chemotactic activity was largely dependent on the epithelial IL-33/CXCL axis, as depletion of *IL-33* in the epithelial cells prior to infection, or treatment of the CM with an antibody cocktail (Neut. Abs) yielded a comparable reduction (**Figure 6A**), although residual activity remained after IL−33 silencing or chemokine neutralization, suggesting that additional factors may also contribute. To model the ability of neutrophils recruited to the cervical mucosa to subsequently adhere to the vascular endothelium during their transmigration from blood to tissue, we assessed dHL−60 adhesion to human endothelial monolayers. As expected, infection-derived CM promoted the stable adhesion of dHL-60 cells to endothelial monolayers, a phenotype that was reversed by upstream *IL-33* silencing (**Figure 6B**). Furthermore, we assessed the cytotoxic potential of the recruited cells. Exposure of dHL-60 cells to *M. genitalium*-CM triggered a potent respiratory burst (**Figure 6C**) and significant release of neutrophil elastase (**Figure 6D**). Importantly, both superoxide generation and degranulation were attenuated to near-basal levels in the si-IL33 and chemokine-neutralization groups. These data demonstrate that the nIL-33-dependent chemokine signature generated during *M. genitalium* infection plays a critical role in orchestrating the recruitment and activation of neutrophil−like cells.

## 4 Discussion

The pathogenesis of *M. genitalium* has long presented an immunological paradox in reproductive medicine. Despite lacking classical exotoxins and often causing only low-grade, persistent infections, this bacterium induces a profound inflammatory response characterized by significant neutrophil infiltration and cervical mucopurulence (14, 41, 42). The current study provides a mechanistic explanation for this phenomenon by demonstrating a non-canonical pathogenic mechanism whereby the pathogen exploits nuclear retention of IL-33 to amplify inflammatory signaling. We have demonstrated that the infection triggers a specific TLR2 and TBK1-dependent signaling axis that drives the transcriptional induction of *IL-33* and its subsequent localization to the nucleus rather than its release as a soluble cytokine. Nuclear IL-33 interacts with the NF-κB p65 subunit and functions as a key transcriptional co-activator, enhancing p65 occupancy at the promoters of the neutrophil-recruiting chemokines CXCL1 and IL-8. The consequent amplification of chemokine secretion provides a molecular explanation for the robust neutrophil infiltration that characterizes the clinical presentation of cervicitis associated with this pathogen.

Cervical epithelial cells are the primary interface for *M. genitalium* infection and function as active sentinels of the mucosal immune system. Upon exposure to the pathogen, these cells utilize pattern recognition receptors to detect bacterial lipoproteins, which serve as potent pathogen-associated molecular patterns (12). Although *M. genitalium* lipoproteins are recognized by TLR, particularly TLR2/6 heterodimers, receptor engagement alone cannot explain the distinct immunopathology of this infection (13, 14). Our findings suggest that the epithelium acts as an immunological relay station. By sensing the pathogen and initiating the nIL-33 cascade, the epithelial cells convert the initial microbial signal into a potent chemotactic gradient. Thus, the epithelium is reprogrammed to sustain inflammation, which may promote chronic disease rather than resolution.

A pivotal finding of this work is the identification of TBK1 as a central mediator of the *M. genitalium*-induced response. Classically, TBK1 is associated with antiviral immunity, functioning downstream of intracellular nucleic acid sensors to phosphorylate IRFs and induce type I interferons (43). The activation of TBK1 by a bacterial lipoprotein agonist suggests that *M. genitalium* infection mimics a pseudo-viral state within the host cell. This activation engages a regulatory pathway in which TBK1 mediates the nuclear translocation of IRF1 and IRF7, which then bind directly to ISREs within the *IL-33* promoter. This discovery addresses a key knowledge gap regarding how IL-33, often considered a constitutively expressed alarmin in other barriers (20), is dynamically regulated at the transcriptional level in the infected endocervix.

The subsequent nuclear accumulation of IL-33 challenges the traditional paradigm of this molecule acting solely as an extracellular alarmin released upon necrotic cell death. In our model, *M. genitalium* infection does not cause immediate cell lysis, yet it induces potent inflammation. We propose that the nuclear retention of IL-33 allows viable, infected cells to function as persistent amplifiers of NF-κB signaling. Prior literature describes nIL-33 as a repressor of NF-κB signaling in certain experimental contexts (25). The basis for this opposite (activator) effect observed in our study remains unresolved, but it likely reflects the highly context−dependent nature of nIL−33 function, influenced by cell type, stimulus, and cell-specific co-regulators. Consistent with an activator role, similar properties of nIL-33 have been documented in endothelial cells, where it functions as a transcriptional regulator of NF-κB p65 to induce cell activation (44). In our endocervical epithelial model, IL-33 depletion is associated with reduced p65 occupancy at the *CXCL1* and *IL-8* promoters. By functioning as a nuclear factor, IL-33 dictates the magnitude of the neutrophil response from within the infected cell and acts as an intrinsic amplifier of the inflammatory cascade.

Our functional assays *in vitro* demonstrate that chemokines dependent on nIL-33 promote the adhesion, chemotaxis, and activation of dHL-60 cells, which serve as a model for neutrophils. Extrapolating to the *in vivo* setting, such a chemotactic gradient could create a steep signal for the recruitment of neutrophils at the infected endocervix. However, the incomplete abrogation of chemotaxis after the knockdown of IL-33 or the neutralization of chemokines implies that other chemoattractants derived from the epithelium (e.g., LTB4, complement components) might also participate in the recruitment of neutrophils during *M. genitalium* infection (45), a possibility that warrants future investigation. If similar mechanisms operate *in vivo*, the recruited neutrophils would release elastase and reactive oxygen species, as observed in our functional assays. Within the reproductive tract, the unmitigated release of these cytotoxic effectors could degrade the extracellular matrix and junctional proteins (46), potentially leading to the disruption of the epithelial barrier. While the recruitment of neutrophils serves to clear the pathogen, persistent activation and subsequent breach of the barrier may provide a molecular rationale for local mucosal damage and sustained cervical inflammation. These latter possibilities remain to be tested in animal models or clinical samples.

The interpretation of these results requires consideration of inherent experimental limitations. First, the End1/E6E7 cell line, while retaining key phenotypic features of primary endocervical epithelium, is immortalized and may exhibit alterations in signaling pathways compared to native tissue. Second, our functional assays relied on dHL-60 cells as a surrogate for human neutrophils. We acknowledge that dHL-60 cells, while a widely used model for neutrophil function, may not fully recapitulate all aspects of primary neutrophil behavior, such as granular enzyme content or life span. Therefore, our functional findings should be considered as surrogate evidence that will need confirmation using primary human neutrophils. Third, our study does not include a three-dimensional tissue architecture or stromal components, which can modulate inflammatory responses. Therefore, our findings establish a mechanistic proof of concept in a simplified system, but their physiological relevance must be validated in more complex models (e.g., organoids, animal models, or biopsies of human tissue).

Limitations regarding inference of pathways also exist. While our data support a functional link between TLR2 and TBK1 in the induction of IL-33, the precise molecular ordering remains to be defined. It is not yet clear whether TBK1 is directly recruited by TLR2 or via intermediate adaptors such as MyD88, IRAK1/4, or TRAF3. Future reconstitution experiments in knockout cell lines or proximity ligation assays will be required to establish direct physical interactions and the hierarchy of signaling events. Furthermore, regarding the induction of IL-33 observed with membrane lipoproteins, we cannot exclude the possibility that heat inactivation may have affected other thermolabile bacterial components, or that the lipoprotein preparation contains minor contaminants that contribute to the effect.

Thus, the present study provides a molecular framework for understanding how *M. genitaliu*m manipulates nIL-33 to amplify neutrophilic inflammation. Future studies using primary endocervical epithelial cells, three-dimensional organoid models, and models of infection in humanized mice will be essential to determine whether this pathway operates *in vivo* and whether targeting nIL-33 or the interaction of nIL-33 with p65 could ameliorate immunopathology. Additionally, single-cell RNA-seq of cervical biopsies from infected women could reveal whether a similar epithelial subpopulation with high IL-33 exists in patients with persistent cervicitis.

## Data Availability

The original contributions presented in the study are included in the article/**Supplementary Material**. Further inquiries can be directed to the corresponding authors.
