# Supplementary material for: Nuclear IL-33 amplifies NF-κB signaling to drive neutrophil recruitment and activation in the endocervical epithelium during Mycoplasma genitalium infection
Source: Front Immunol. 2026 Jul 22;17:1820874. doi: 10.3389/fimmu.2026.1820874 (PMC13437461; doi:10.3389/fimmu.2026.1820874)
Supplement: Supplementary file 1 [file DataSheet1.docx]

**Yu et al. Supplemental Materials**

**Table S1. Primer sequences and amplicon information for ChIP‑qPCR.**

| Target gene | Primer name | Sequence (5′→3′) | Amplicon length  (bp) | Amplicon coordinates (relative to TSS) | Purpose |
| --- | --- | --- | --- | --- | --- |
| *IL-33* promoter | Forward | TGCTCAGGTTTGGGGATGATT | 152 | –491 to –340 | ISRE (IRF1/IRF7 binding) |
| *IL-33* promoter | Reverse | TGAGACAGCAACAGATGGCT | 152 | –491 to –340 | ISRE (IRF1/IRF7 binding) |
| IL-8 promoter | Forward | AAGTGTGATGACTCAGGTTTGC | 135 | –145 to –11 | NF‑κB (p65 binding) |
| IL-8 promoter | Reverse | GTGCTCCGGTGGCTTTTTAT | 135 | –145 to –11 | NF‑κB (p65 binding) |
| CXCL1 promoter | Forward | TCGATCTGGAACTCCGGGAA | 85 | –94 to –10 | NF‑κB (p65 binding) |
| CXCL1 promoter | Reverse | AGATCCGCGAACCCCTTTTA | 85 | –94 to –10 | NF‑κB (p65 binding) |
| Negative control | Forward | TGGCTGTTACTTGTCACCCC | 122 | -615 to -494 | Background control |
| Negative control | Reverse | GGCTATGTCTGCCCTTTGGA | 122 | -615 to -494 | Background control |

**Fig. S1.** ***M. genitalium* persists in association with End1/E6E7 cells.** End1/E6E7 cells were infected with *M. genitalium* (MOI 100), and at 0, 12, 24 and 48 h post-infection monolayers were washed three times with PBS to remove non-adherent organisms. Cell-associated *M. genitalium* was quantified by qPCR targeting *mgpB* and is expressed as *mgpB* genome copies per 10⁵ host cells. Data are mean ± SEM of three independent biological experiments (n = 3); ns, no significant difference among the 12, 24 and 48 h time points (one-way ANOVA with Tukey's post-hoc test).

**Fig. S2. Assessment of subcellular fractionation purity.** Representative immunoblots of nuclear (Histone H3) and cytoplasmic (GAPDH) markers in whole cell lysates (WCL), nuclear fractions (N), and cytoplasmic fractions (C) from End1/E6E7 cells. Densitometric quantification of three independent fractionation experiments. For Histone H3, the percentage of total signal detected in the nuclear fraction was (96.47 ± 0.405)%, and in the cytoplasmic fraction was (3.05 ± 0.586)%. For GAPDH, the percentage of total signal detected in the cytoplasmic fraction was (96.5 ± 0.574)%, and in the nuclear fraction was (3.250 ± 0.278)%.

**Fig. S3. *M. genitalium* infection at MOI 100 does not induce significant cytotoxicity in End1/E6E7 endocervical epithelial cells.** End1/E6E7 cells were either mock‑infected (treated with sterile SP‑4 medium without bacteria, following the same centrifugation and incubation procedures as the infected groups) or infected with *M. genitalium* at MOI 50 or 100 for 24 h. Cell‑free culture supernatants were collected, and LDH activity was measured using a CyQUANT LDH Cytotoxicity Assay kit following the manufacturer’s instructions (Thermo). Cells lysed with 1% Triton X‑100 were used as positive control (100% LDH release). Cytotoxicity was calculated as: (Experimental OD – Mock OD) / (positive control OD – Mock OD) × 100%. Data represent mean ± SEM from three independent experiments (n=3). ns, not significant (one‑way ANOVA with Dunnett’s post-hoc vs. Mock).

**Fig. S4. *M. genitalium* membrane lipoprotein induces TBK1 phosphorylation in a TLR2-dependent manner.** End1/E6E7 cells were left unstimulated (control) or stimulated with purified *M. genitalium* membrane lipoprotein (LP, 10 μg/mL), with or without pretreatment with the TLR2 inhibitor C29 (50 μmol/L, 1 h). After 30 min, phosphorylation of TBK1 at Ser172 (p-TBK1) was assessed by immunoblot; total TBK1 served as the loading control. Densitometric quantification of phosphorylated TBK1 levels (normalized to total TBK1) from three independent experiments is presented (right panels). Data are mean ± SEM. **p < 0.01 (one-way ANOVA with Dunnett's post-hoc test).

**Fig. S5. Host-directed inhibitors and knockdowns do not alter cell-associated *M. genitalium* burden.** End1/E6E7 cells were treated with the indicated inhibitors or transfected with the indicated siRNA/shRNA and then infected with *M. genitalium* (MOI 100) under the same conditions used for the corresponding signaling experiments. At 24 h post-infection, monolayers were washed three times with PBS and cell-associated *M. genitalium* was quantified by *mgpB* qPCR (genome copies). (A) Infection alone versus infection in the presence of the TLR2 inhibitor C29, the TBK1 inhibitor BX795, or the TLR4 inhibitor TAK-242. (B) Infection with non-targeting control siRNA (si-NC) versus TBK1 siRNA (si-TBK1) or IL-33 siRNA (si-IL33). (C) Infection with non-targeting control shRNA (sh-NC) versus IRF1 shRNA (sh-IRF1) or IRF7 shRNA (sh-IRF7). Data are mean ± SEM of three independent biological experiments (n = 3); ns, no significant difference versus the corresponding infected control (one-way ANOVA with Dunnett's post-hoc test).

**Fig. S6. TBK1 knockdown attenuates IRF1 and IRF7 nuclear accumulation during *M. genitalium* infection.** End1/E6E7 cells were transfected with control siRNA (si-NC) or TBK1‑specific siRNA (si-TBK1) for 48 h, followed by infection with *M. genitalium* (MOI 100, 24 h). Nuclear fractions were isolated and analyzed by immunoblotting for IRF1, IRF7, and Histone H3 (nuclear loading control). Representative blots from three independent experiments are shown (left panels). Densitometric quantification of nuclear IRF1 and IRF7 levels (normalized to Histone H3) from three independent experiments is presented (right panels). Data are mean ± SEM. *p < 0.05 (unpaired t‑test vs. siNC).

**Fig. S7. Validation of IRF1 and IRF7 knockdown efficiency by shRNA.** End1/E6E7 cells were transfected with scrambled control shRNA (sh-NC) or shRNA targeting IRF1 (sh-IRF1) or IRF7 (sh-IRF7) for 48 h. Whole‑cell lysates were subjected to immunoblotting with antibodies against IRF1 (A), IRF7 (B), and β-actin (loading control). Representative blots from three independent experiments are shown. Densitometric quantification of band intensities from three independent experiments is presented as relative protein levels normalized to β-actin. Data are mean ± SEM. *p < 0.05 (unpaired t‑test vs. sh-NC).

**Fig. S8. Infection-induced nuclear IL-33 is full-length.** Nuclear fractions from uninfected and *M. genitalium*-infected (MOI 100, 24 h) End1/E6E7 cells were immunoblotted with an N-terminal-specific (N-term, aa 1–111, R&D Systems, AF4810) or a C-terminal-specific (C-term, aa 112–270, R&D Systems, MAB3625) anti-IL-33 antibody in separate experiments, each with Histone H3 as the nuclear loading control. Both antibodies detected a single band at ~31 kDa in the infected nuclear fraction, consistent with full-length IL-33 and clearly distinct from the ~18–21 kDa mature/cleaved form; a recombinant full-length human IL-33 standard (FL-rhIL-33, aa 1–270, LSBio, LS-G14580) was run for size reference. Data are representative immunoblots.

**Fig. S9. Validation of nIL-33 knockdown by siRNA at the protein level.** End1/E6E7 cells were transfected with control siRNA (si-NC) or IL‑33‑specific siRNA (si-IL‑33) for 48 h, followed by infection with *M. genitalium* (MOI 100, 24 h). Nuclear fractions were isolated and subjected to immunoblotting with antibodies against IL‑33 and Histone H3 (nuclear loading control). Representative blots from three independent experiments are shown. Densitometric quantification of nuclear IL‑33 levels (normalized to Histone H3) from three independent experiments is presented. Data are mean ± SEM. *p < 0.05 (unpaired t‑test vs indicated group).
